# Using mechanistic models to highlight research priorities for tick-borne zoonotic diseases: Improving our understanding of the ecology and maintenance of Kyasanur Forest Disease in India

**DOI:** 10.1371/journal.pntd.0011300

**Published:** 2023-05-01

**Authors:** Richard M. J. Hassall, Sarah J. Burthe, Stefanie M. Schäfer, Nienke Hartemink, Bethan V. Purse

**Affiliations:** 1 UK Centre for Ecology & Hydrology, Wallingford, United Kingdom; 2 UK Centre for Ecology & Hydrology, Edinburgh, United Kingdom; 3 Biometris, Wageningen University and Research, Wageningen, The Netherlands; 4 Quantitative Veterinary Epidemiology Group, Wageningen University and Research, Wageningen, The Netherlands; Beijing Children’s Hospital Capital Medical University, CHINA

## Abstract

The risk of spillover of zoonotic diseases to humans is changing in response to multiple environmental and societal drivers, particularly in tropical regions where the burden of neglected zoonotic diseases is highest and land use change and forest conversion is occurring most rapidly. Neglected zoonotic diseases can have significant impacts on poor and marginalised populations in low-resource settings but ultimately receive less attention and funding for research and interventions. As such, effective control measures and interventions are often hindered by a limited ecological evidence base, which results in a limited understanding of epidemiologically relevant hosts or vectors and the processes that contribute to the maintenance of pathogens and spillover to humans. Here, we develop a generalisable next generation matrix modelling framework to better understand the transmission processes and hosts that have the greatest contribution to the maintenance of tick-borne diseases with the aim of improving the ecological evidence base and framing future research priorities for tick-borne diseases. Using this model we explore the relative contribution of different host groups and transmission routes to the maintenance of a neglected zoonotic tick-borne disease, Kyasanur Forest Disease Virus (KFD), in multiple habitat types. The results highlight the potential importance of transovarial transmission and small mammals and birds in maintaining this disease. This contradicts previous hypotheses that primates play an important role influencing the distribution of infected ticks. There is also a suggestion that risk could vary across different habitat types but currently more research is needed to evaluate this relationship. In light of these results, we outline the key knowledge gaps for this system and future research priorities that could inform effective interventions and control measures.

## Author summary

The risk of humans contracting zoonotic diseases (diseases passed from animals to humans) is being altered by changes in the environment. These changes are occurring rapidly in tropical areas, which are burdened with neglected diseases that often disproportionately affect poorer communities. These diseases generally receive little attention and are less well studied. This means we often have a limited understanding of the ecological processes that influence the risk of people catching zoonotic diseases, which can be crucial in designing effective interventions. In this study, we developed mathematical models to highlight the ecological processes that influence the maintenance of Kyasanur Forest Disease, a neglected zoonotic disease affecting people in India. Using this approach, we were able to determine the hosts and forms of transmission that are most likely to play important roles in maintaining this pathogen and understand how risk to humans might vary in different habitats. We were also able to highlight key knowledge gaps and future research priorities that would help to inform interventions. An additional benefit of this approach is that it can also be used for other tick-borne diseases to help understand how pathogens are maintained and to prioritise the research questions that need to be addressed in other disease systems.

## Introduction

Many zoonotic diseases have complex transmission cycles involving communities of vectors and animal hosts, with heterogeneity in disease dynamics being dependent on host and vector ecology and evolutionary biology [1–4]. To understand and predict human spillover of zoonotic diseases, this heterogeneity in disease dynamics also needs to be considered in conjunction with human behaviour and use of ecosystems which can determine exposure to infected vectors and hosts [5,6]. The design and implementation of effective disease control is often limited by a poor ecological evidence base, specifically by lack of knowledge of which different vector and host species, and which processes, are contributing to transmission and human spillover [4,7]. This is particularly the case for neglected zoonotic diseases that primarily affect poor and marginalised populations in low-resource settings [8], in which less attention and funding is available for research and interventions [4,9,10].

New frameworks have been put forward, which aim to prevent and manage zoonotic diseases by identifying the hierarchical series of barriers that must be overcome by a pathogen to facilitate spillover from an animal reservoir into a human or livestock host [7,11,12]. Given improved ecological knowledge, these barriers, such as sufficient density and competence of reservoir hosts, can be targeted by ecological interventions complementing more conventional human-centred interventions such as vaccination [7,12]. In such frameworks, it needs to be taken into account that vector and reservoir roles will depend on the ecosystems in which species are embedded and the relative contributions to transmission of other species within the community [13]. Several global change drivers, including land use change, agricultural intensification and human settlement, are hypothesised to be contributing to zoonotic disease spillover by bringing people into greater contact with domestic and wildlife animal reservoirs and vectors at the interfaces between human habitation, agricultural and natural habitats [11,14–16]. Human modified landscapes have been shown to support an increased diversity of zoonotic hosts [17] and could result in changes in host densities and interspecies contact rates altering disease dynamics [18]. Such zones of human-animal-environment interfaces or ecotones now dominate much of the land in tropical regions where the burden of neglected zoonotic diseases is highest and land use change and forest conversion is occurring most rapidly [16]. Within such settings, attempts to build the ecological evidence base to inform interventions must take account of potential variation in host and vector roles and their habitat associations and interactions that govern transmission processes.

Kyasanur Forest Disease (KFD) is one example of a neglected zoonotic disease for which we still have a poor ecological evidence base [7]. The causal agent, Kyasanur Forest Disease Virus, is a tick-borne flavivirus causing potentially fatal haemorrhagic disease in people in the Western Ghats region of south India, with 400–500 reported cases a year and mortality rate of up to 10% [19]. Humans can contract KFD when bitten by an infected tick but are considered dead-end hosts for the disease [20]. The disease primarily affects rural forest communities, including tribal groups and plantation and forestry workers [21–23]. Approximately 69% of small-holder farmers and tribal groups surveyed in the region have reported that they were concerned by the impact KFD has had on their livelihoods, highlighting the impact of this disease in the region [22,24].

KFD has a complex transmission cycle in which various tick species (mostly *Haemaphysalis* genus, but also some *Ixodes*) and vertebrate hosts, (including rodents and shrews, monkeys and birds), have been implicated [25]. Monkeys, mainly the black-footed grey langur (Se*mnopithecus hypoleucos*) and the bonnet macaque (*Macaca radiata*), are hypothesised to act as amplifying hosts, by infecting large numbers of larval ticks with the virus, and are believed to create a hotspots of infections when they die [26]. However, empirical evidence to support this hypothesis is lacking and there is very limited recent information on the potential role of other relevant wild hosts such as small mammals and birds following substantial land use change [7]. To date there is also a very poor understanding of the importance of transovarial transmission in the maintenance of KFD, where infection is passed from an adult female to their eggs. Although transovarial transmission has been demonstrated in laboratory studies in some native Indian *Ixodes* tick species such as *Ixodes petauristae* [27,28] there is no evidence of this occurring in *Haemaphysalis* ticks in situ [7]. If ticks are able to maintain KFD through transovarial transmission this could add significance to the potential role of cattle in this system, which regularly graze in forests and a variety of other habitat types also used by small mammals, birds and primates. Cattle do not act as a hosts for systemic transmission, due to the fact that they do not develop a long-lasting viraemia [29,30], but may amplify tick populations as an important blood meal host and may influence the distribution of infected ticks [31,32]. Currently, empirical knowledge of the role of different species of vector and hosts in the distribution of infected ticks and transmission cycle of KFD is lacking but management guidelines advocate for tick control on cattle and around the sites of host (monkey) deaths, despite limited empirical evidence to support these strategies [7].

Correlative modelling of recent human outbreaks indicates that the risk of virus spillover into humans is highest in diverse agro-forestry landscapes, created when moist evergreen forest is replaced with plantations and paddy cultivation [33], consistent with the hypothesis that KFD is an ecotonal disease [25]. These agro-forest mosaics can consist of a number of different habitat types but it remains unclear how variation in host densities and tick abundance and burden on hosts across these different habitats might influence the maintenance and persistence of KFD and subsequently exposure of people using these landscapes.

Models have a key role to play in predicting the variable impact of hosts and vectors on disease dynamics and infection risk across human-animal-environment interfaces and rapidly changing landscapes [4,15] but need to be contextualised carefully to local empirical data availability and knowledge needs for the disease interventions [34]. Models are particularly critical for understanding tick-borne disease systems (TBDs), in which ticks take one blood meal per life stage and may feed on and transmit infection to different vertebrate hosts at different life stages (larva, nymph and adult) via different routes of transmission. These include transovarial transmission between adult ticks and eggs, non-systemic transmission between ticks co-feeding on the same hosts and systemic transmission between infected hosts and larval, nymph or adult ticks. A number of modelling approaches have been used to understand and compare the relative contribution of different transmission routes and to explore the effects of tick and host demography and diversity on pathogen persistence [35–37]. However, they have rarely been explicitly linked to local management strategies. In their Resource-Based Habitat Concept for vector-borne diseases, Hartemink et al. [38] advocated for integrating functional resource use of each host, pathogen and vector species, linked to particular habitats across landscape mosaics into spatial predictive frameworks (see also Vanwambeke et al. [39]). Though some models have incorporated host habitat use and tick-host interactions, these are largely confined to temperate, resource-rich settings and systems such as Lyme disease and tick-borne encephalitis in the United States and Europe [40–42]. For neglected zoonotic disease systems, mechanistic modelling approaches that combine available empirical data with knowledge and data from other similar disease systems can enable insights into the key processes that may contribute to the maintenance of pathogens. Collecting empirical data can be logistically challenging and costly, and by exploring the sensitivity of models to different parameters it is possible to highlight parameters for which robust empirical estimates would reduce uncertainty in predictions whilst also identifying parameters that are less likely to be influential [43]. This can aid in framing key knowledge gaps and potential priorities for future research and interventions.

Given KFD’s high human health impact, with spillover sensitive to land use change, low ecological evidence-base for interventions and complex transmission cycle, it is an ideal and important test case with which to explore the insights into host roles, transmission processes and habitat associations that can be gained by combining mechanistic models with extant empirical data and local management needs. Here, we utilise a modelling approach to better understand the relative contribution of different host groups (small mammals, birds and primates) and transmission routes to the maintenance of this pathogen. The former represent priority local knowledge needs to inform improved interventions [7]. We also investigate how the maintenance and persistence of the KFD virus may vary in different habitat types in agro-forest mosaics where spillover tends to occur. To achieve this, we used next generation matrix (NGM) approaches for tick-borne pathogens already developed by Hartemink et al. [43]. Previous NGM approaches investigating KFD have estimated the contribution of different modes of transmission to the *R*_0_ of KFD [44], but have so far considered five ‘types-at-infection’; ticks infected at different life stages (egg, larva,nymph and adult) and newly infected vertebrate hosts as a single host type. To further understand the contribution of different vertebrate hosts we build on previous models by expanding the number of host types to include small mammals, birds and primates as individual ‘types-at-infection’ as well as adapting this framework to incorporate the densities of different vertebrate hosts. This will allow us to assess the likely contribution of different host groups to systemic transmission of KFD as well as the relative contribution of transovarial transmission and non-systemic (co-feeding) transmission in maintaining KFD. In addition, we also consider the potential changes in *R*_*0*_ and reservoir host roles for KFD across different habitat types by introducing scaling factors to account for potential variation in tick abundance and subsequent burdens on hosts as a result of different cattle densities or habitat types. Using the results from these models, we aim to highlight the knowledge gaps and future research priorities that would help to improve our understanding of natural foci of KFD and aid in the development of interventions.

## Methods

### Next generation matrix model for KFD

The NGM approach used here provides an intuitive estimate of *R*_*0*_ for tick-borne pathogens, where *R*_0_ is derived as the largest eigenvalue of the NGM [45]. This eigenvalue is indicative of pathogen generations growing in size when it is greater than 1 and declining in size when the eigenvalue is less than 1 as well as providing an estimate of the per generation increase in the number of infected hosts or tick life stages. This approach also allows the relative contribution of different transmission routes to *R*_0_ to be established [43,44]. The NGM model includes 7 types-at-infection: (1) Tick-infected-as-egg, (2) Tick-infected-as-larva, (3) Tick-infected-as-nymph, (4) Tick-infected-as-adult, (5) Newly-infected-small-mammal, (6) Newly-infected-bird, (7) Newly-infected-primate.

**Fig 1:**
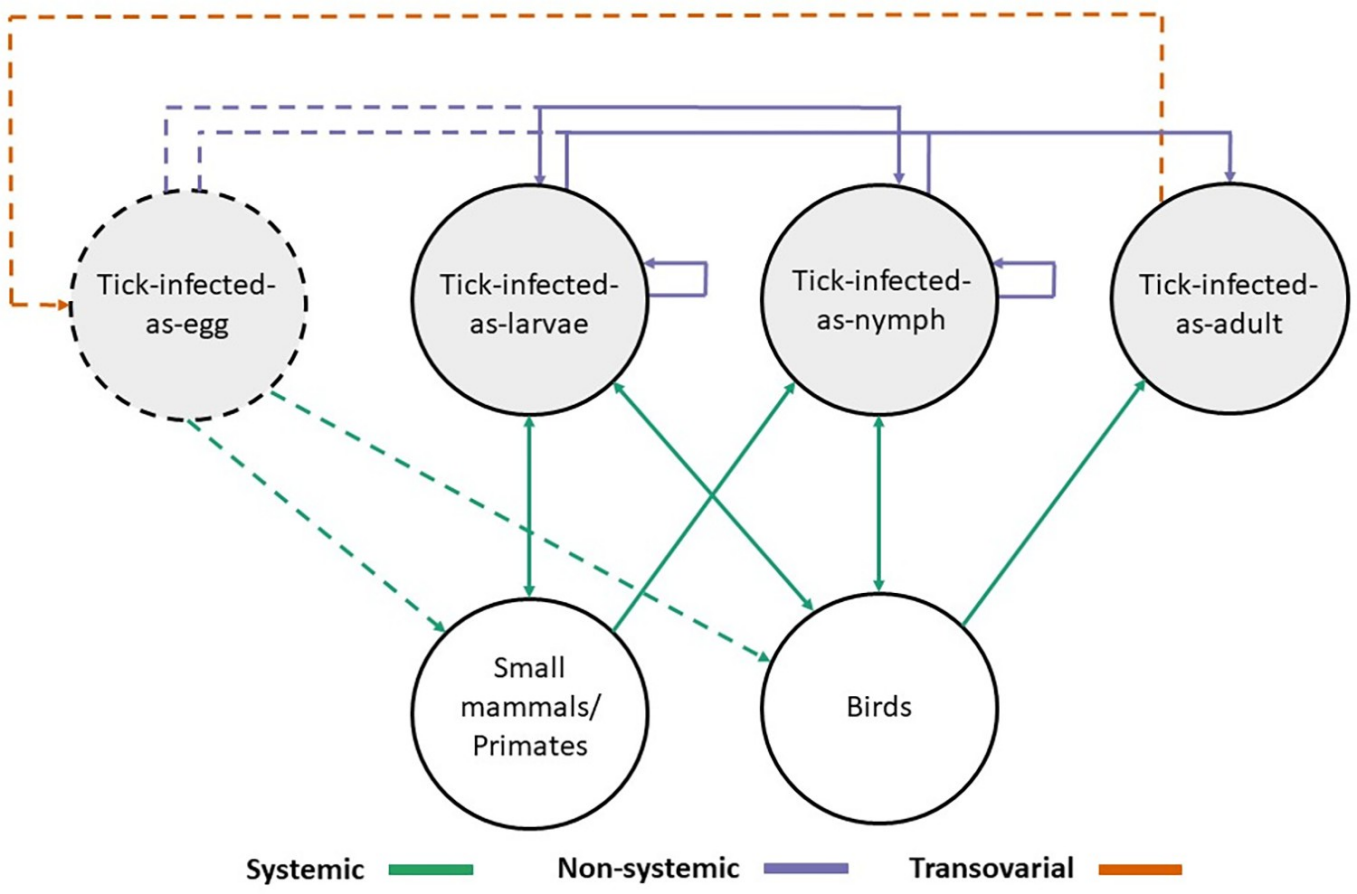
Transmission graph for the NGM model. The edges and arrows indicate the transmission pathways that can generate infected individuals. These are grouped into three main categories. 1) Systemic transmission: Vertebrate hosts are infected by infected ticks and can produce ticks infected at different life stages (it is assumed that adult ticks do not to feed on small mammals or primates). 2) Non-systemic transmission: infected ticks can infect other ticks of different life stages by feeding in close proximity. 3) Transovarial transmission: Infected adult ticks can pass infection onto their eggs producing ticks infected-as-eggs. Transovarial transmission and all other transmission pathways that can arise from it are represented by dashed lines. When transovarial transmission is removed from the models these pathways of transmission are removed.

Given the uncertainty around transovarial transmission of KFD occurring in the wild [7], we included a second NGM model excluding the possibility for transovarial transmission and the tick infected as egg type-at-infection. This allowed us to consider the potential impact of transovarial transmission on the persistence of KFD. Both models included the assumption that adult ticks do not feed on primates and small mammals, based on previous data from Rajagopalan et al. [46] and Trapido et al. [47]. Therefore, infected small mammals and primates can infect only larvae and nymphs (thereby producing new cases of type-at-infection 2 and 3, respectively, ticks-infected-as-larvae and ticks-infected-as-nymphs). Within the model, ticks take one blood meal per life stage, and when they get infected, they can only pass this infection on in the next life stage. This, combined with the fact that adults do not feed on small mammals and primates, means that these hosts can only be infected by ticks that were infected as an egg (during the bloodmeal they take as larva or nymph) or by ticks that were infected as a larva (during their bloodmeal as nymph). For the conceptual model see Fig 1.

To adapt the model framework to account for different vertebrate host types we extended the equations presented in Hartemink et al. [43]. In this earlier model, the *H*_*c*_ parameter served as a proxy for the probability that a tick will bite a host suitable for transmission. In this model, we included an additional parameter *Pb*_*i*_ as a proxy for the probability that a tick bite is on a specific competent host *i*, which is proportional to the proportion of the competent host community that consists of host *i*. This parameter was also made life stage specific to account for the assumption that adult ticks do not feed on small mammals or primates [46,48]. For example, the number of birds infected by a tick that was infected as a nymph would be described by Eq 1:

$$k_{6,3}=(S_{a}QaH_{c})\;{PbA}_{bir}$$ (Eq 1)

where, *S*_*a*_ is the probability of a nymph surviving to become an adult and biting any host, *Qa* is the probability of transmission, given that an adult tick bites a host and *PbA*_*bir*_ is the probability that an adult tick will bite a bird. The latter is based on the relative host density of birds and the preference of the relevant tick life stage to bite a bird.

By explicitly considering each host, we were then able to calculate the relative contribution of each host to systemic transmission. As outlined in Matser et al. [44], the elasticities of *R*_0_ to the individual matrix elements (or proportional change in *R*_0_ in response to a proportional change in each matrix element) can be interpreted as the relative contribution of a matrix element to *R*_0_. Therefore, the relative contribution of systemic transmission to *R*_0_ can be calculated by summing the elasticities of matrix elements associated with all systemically infected hosts to establish the composite elasticity of these elements [43,44]. In this framework, each host is associated with its own matrix elements, this allowed the relative contribution of each vertebrate host to systemic transmission to be determined by calculating the composite elasticities of matrix elements associated with each individual host and dividing this by the relative contribution of systemic transmission to *R*_0_.

### Scenarios

Using this NGM framework, we outlined different scenarios to see how *R*_0_ and the contribution of different transmission pathways and vertebrate hosts to *R*_0_ may change in different habitat types with different host community compositions. To do this we focused on five different relevant habitat types present in the agroforestry mosaic where KFD transmission occurs [25,33]: evergreen forest, deciduous forest, agriculture and plantations (excluding paddy), paddy and areas of human habitation.

We aimed for our models to reflect the fact that tick densities may vary between areas, e.g. as a result of different habitat or different availability of hosts. Tick densities may affect tick burdens on hosts, as well as the number of eggs produced by female ticks. To account for factors influencing tick abundance in the environment and tick burden on hosts, we used two approaches, namely two different scaling factors to reduce or increase the number of eggs produced by adult females and tick burdens on hosts, and we compared the results. These scaling factors influenced the number of tick-infected-as-egg produced as a result of transovarial transmission, as well as the number of each life stage present and co-feeding on hosts.

For the first scaling factor, we assumed that tick abundance and burden will depend solely on habitat type as result of availability of hosts and environmental factors that may influence the survival of ticks. This was modelled by applying a scaling factor that accounts for variation in tick densities in different habitat types, which was calculated as one minus the proportion decrease in tick density in relation to the habitat type with the highest tick density. It was applied to the number of eggs produced by adult females and to all parameters related to tick burden (Eq 2). To estimate the change in tick abundance across habitat types we used data on the difference between numbers of nymphs collected in different land use types in Europe [49]. This provides some estimate of how ticks may be distributed across habitat types in general but it is worth noting that agricultural practices and plants used in crops and plantations will differ and the focal area in India has a greater number of forest types being in a tropical region. The reason for using data from Europe was due the lack of availability of local data on the distribution of *Haemaphysalis* ticks across different land use types.

As outlined previously, cattle freely graze across multiple habitat types in this system and increased densities of cattle are hypothesised to result in an increased abundance of ticks [31,32]. We modelled this using a second scaling factor to account solely for the potential influence of cattle density on tick abundance and burden. Currently, there are no data available from India on the rate of increase in tick abundance as a result of increased cattle density. This relationship has been quantified for large wild ungulates in Europe and given a lack of data we used results from a different disease system that outlines the rate of increase in the density of questing nymphs in relation to the density of deer (0.026) [50]. As above, this scaling factor was applied to the number of eggs produced by adult females and to all parameters related to tick burden (Eq 3).

Cattle is not a type-at-infection in the matrix model, as it is a dead end host for KFD virus. It was however included as part of the matrix elements associated with non-systemic transmission, to account for the fact that ticks feeding in close proximity on cattle may be able to infect each other. For example, the formula for the matrix element quantifying the number of adult ticks infected by a tick that was infected as a nymph through non-systemic transmission is described by Eq 2 (using scaling factor calculated using habitat type) and Eq 3 (using scaling factor calculated using cattle density):

$$k_{4,3}=S_{a}H_{c}({N_{p}Caa}_{cat}C_{s}\theta_{TT}{Pb}_{cat}+{N_{p}Caa}_{pri}C_{s}\theta_{TT}{Pb}_{pri}+{N_{p}Caa}_{bir}C_{s}\theta_{TT}{Pb}_{bir}+{N_{p}Caa}_{sm}C_{s}\theta_{TT}{Pb}_{sm})$$ (Eq 2)

$$k_{4,3}=S_{a}H_{c}({(L}_{d}r_{L}+1){Caa}_{cat}C_{s}\theta_{TT}{Pb}_{cat}+{(L}_{d}r_{L}+1){Caa}_{pri}C_{s}\theta_{TT}{Pb}_{pri}+{(L}_{d}r_{L}+1){Caa}_{bir}C_{s}\theta_{TT}{Pb}_{bir}+{(L}_{d}r_{L}+1){Caa}_{sm}C_{s}\theta_{TT}{Pb}_{sm})$$ (Eq 3)

Where *Pb*_*sm*_, *Pb*_*pri*_, *Pb*_*bir*_ and *Pb*_*cat*_ outline the proportion of the host community competent for non-systemic transmission that is made up of small mammals, primates, birds and cattle respectively. *θ*_*TT*_ is the efficiency of transmission from tick to tick and *Caa*_*i*_ is the number of adult ticks expected to be feeding with a single adult tick on each host species. *L*_*d*_ and *r*_*L*_ (Eq 2) are the density of cattle and the rate of increase in tick burden respectively. *N*_*p*_ is one minus the proportion of decrease in tick abundance in relation to the habitat type with the highest tick abundance.

### Host community composition in different habitat types

For each habitat type, we varied parameters within the model to reflect potential habitat-specific differences in host community composition. Data on host community composition in each habitat type is not available at this time from specific KFD affected areas within the Western Ghats. In our study, we were able to source data from studies carried out in Western Ghats that report the number of animals recorded or trapped and the area covered [51–54]. We used these data to represent the small mammal, bird, primate and cattle densities in different habitat types in KFD affected areas.

For small mammals, Molur & Singh [52] recorded the number of individuals captured and the area (ha) for 14 rodent species in evergreen forest, deciduous forest, agricultural land (including ginger and paddy), plantations (banana, cardamom, coffee, orange and tea), and areas of human habitation. We used these data, and, we scaled the observed number of individuals trapped per hectare up to the number of individuals trapped per 1 km^2^ in evergreen and deciduous forest, agriculture (including paddy) and human habitation habitat types. We also used this method for plantation habitat types: banana, cardamom, coffee, orange and tea, and calculated the mean number of individuals trapped per 1 km^2^ to estimate small mammal density in plantations.

For birds, Pramod et al. [53] outlined data for number of birds in evergreen forest, deciduous forest, plantation, paddy and areas of human habitation in the Western Ghats. Abundance data for birds was collected in 600m x 100 m belt transects in different habitat types for 212 bird species [53,55]. We scaled this data up to individuals recorded per km^2^ assuming transects covered an area of 0.06 km^2^. As a proxy for bird density in evergreen forest, we used the middle of the range of densities of birds per km^2^ in both the evergreen forest and semi-evergreen forest habitat types. For deciduous forest, we used data obtained from the moist and dry deciduous forests habitat types. Data was also available for individuals recorded in paddy fields, monoculture plantations habitat types as well as the garden and habitation habitat type. To refine our estimates of bird densities we focused on 14 bird species that tend to live or forage on the ground and which tended to have the highest burden of ticks in KFD areas [56]. This included babblers, thrush, mynas, crow pheasant and junglefowl (See Table C in S1 Text for list of focal species). We utilised bird detection frequency data from eBird [57] for Karnataka in India and calculated the sum of the mean detection frequency for all species and the proportion of that made up by our focal bird species. We then scaled bird density estimates based on this proportion.

For primates, Bapureddy et al. [54] report estimated densities of individuals per km^2^ of the gray langur and bonnet macaque in evergreen and deciduous forest types at 12 locations. Using these data we calculated the average densities at sites where habitat types were deciduous forest type or evergreen forest. Singh and Rao [58] report the number of bonnet macaques per km of occupied area in intensive agriculture (including paddies) and dry agriculture (including plantations) for three years (1989, 1998 and 2003) and we used the mean across the three time periods to determine the density of primates in these habitat types.

As a proxy for cattle densities we used the mean indigenous cattle density across the Shimoga region in Karnataka (60 cattle per km) [33] and adjusted this using recently collected data on habitat use of indigenous cattle in Shimoga. The Ashoka Trust for Research in Ecology and the Environment (ATREE) and the Monkey Fever Risk project (monkeyfeverrisk.ceh.ac.uk) shared this data with us (Table D in S1 Text). These cattle are free to move across multiple habitat types during the day and this data on cattle movement outlines the average proportion of time spent in a habitat type vs the proportion of area made up of that habitat type, 0.67 (evergreen forest), 1.99 (deciduous forest), 1.14 (plantation) and 0.74 (cropland). Therefore, we scaled density to reflect the ratio of the proportion of time spent in each habitat type vs the proportion of habitat available in the area and reduced density where the ratio was less than one: 40.2 cattle per km (evergreen forest), 60 cattle per km (deciduous forest), 60 cattle per km (plantation) and 44.4 cattle per km (cropland).

The densities calculated above were used as a proxy for the number of hosts per 1 km^2^ and proportion of the total host community that each host makes up (Table 1).

**Table 1:** Table showing the proportion of host community made up of small mammals (Pb_sm_), birds (Pb_bir_), primates (Pb_pri_) and cattle (Pb_cat_) in each habitat type, which serves as a proxy for the probability that a tick will bite each host. Numbers in brackets show estimated density (individuals per km^2^).

Parameter	Evergreen forests	Deciduous forests	Agriculture /plantation	Paddy	Human habitation
*Pb*_*sm*_	0.922 (1537.65)	0.947 (2891.56)	0.913 (1283.42)	0.933 (1760)	0.92 (2315.78)
*Pb*_*bir*_	0.045 (74.93)	0.030 (93.06)	0.041 (57.53)	0.04 (75.24)	0.046 (116.6)
*Pb*_*pri*_	0.009 (15.25)	0.008 (25.6)	0.004 (5.23)	0.003 (5.86)	0.010 (25.6)
*Pb*_*cat*_	0.024 (40.1)	0.020 (60)	0.043 (60)	0.024 (44.4)	0.024 (60)

We performed 100 runs of the model for each habitat type using cattle density as a scaling factor and 100 runs of the model for each habitat type using habitat type as a scaling factor.

For all equations and parameters used in the model, see S1 Text.

### Sensitivity and elasticity analysis

To assess how important individual parameters are when estimating *R*_0_, we calculated the sensitivity and elasticity of *R*_0_ to each individual parameters used in the equations for matrix elements. Sensitivity values represent the rate of change in *R*_0_ given an incremental change in an individual parameter and elasticity values represent a proportional change in *R*_0_ given a proportional change in an individual parameter. If both sensitivity and elasticity values are high for a parameter then small changes in this parameter can result in relatively large changes in *R*_0_ [43]. For each run, the sensitivity of *R*_0_ to changes in individual parameters (*S*) was calculated using the equation defined in Caswell [59].


$$S=\sum_{all\;elements\;with\;a}\frac{\partial k_{ij}}{\partial a}\frac{\partial R_{0}}{\partial k_{ij}}$$

With $\frac{\partial k_{ij}}{\partial a}$ representing the change of the focal element (*k*_*ij*_) with respect to the focal parameter *a* multiplied by the sensitivity of *R*_0_ to the focal element (*k*_*ij*_). The sum of these values for all matrix elements derived using the focal parameter (*a*) gives the sensitivity of *R*_0_ to each individual parameter. The elasticity can then be calculated by $S\frac{a}{R_{0}}$.

## Results

**Fig 2:**
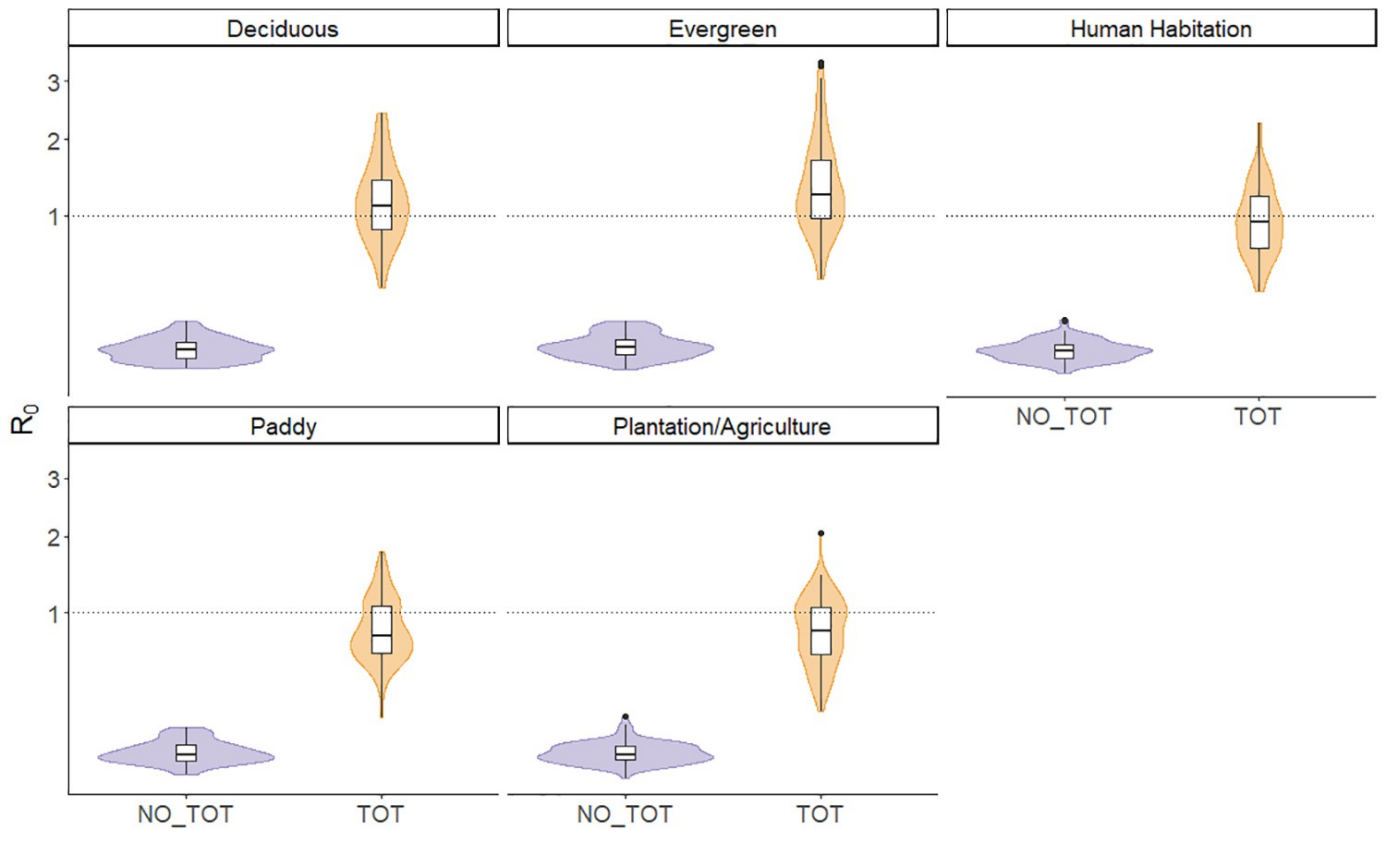
Distribution of values in each habitat type for models using scaling factor based on habitat type. Plot shows results for models excluding transovarial transmission (NO_TOT) and including transovarial transmission (TOT). For results using cattle density as scaling factor see Fig A in S2 Text. Coloured density kernels around boxplots show the distribution of R_0_ estimates from all model runs.

We performed 100 runs for each scenario with the model using cattle density as a scaling factor (“cattle model”) and the model using habitat type as a scaling factor (“habitat model”), both including and excluding transovarial transmission. The results of these models show that in all scenarios, the models excluding transovarial transmission have a *R*_*0*_ of less than one in most runs when habitat type (Mean *R*_*0*_ = 0.071, SD = 0.035) and cattle density (Mean *R*_*0*_ = 0.135, SD = 0.06) were used as scaling factors (Fig 2 and Fig A in S2 Text). When transovarial transmission is included in the model using cattle density to scale tick abundance and burden, *R*_*0*_ was generally similar and consistently exceeded one in all scenarios (Mean *R*_*0*_ = 1.83, SD = 0.789) (Figs A and B and Table A in S2 Text)). However, when tick abundance and burden is scaled based on the habitat type, there was more variation in *R*_*0*_, which was generally below one in the human habitation, paddy and plantation and above one in evergreen forest and deciduous forest (Mean *R*_*0*_ = 1.05,SD = 0.49) (Fig 2 and Fig C and Table B in S2 Text).

**Fig 3:**
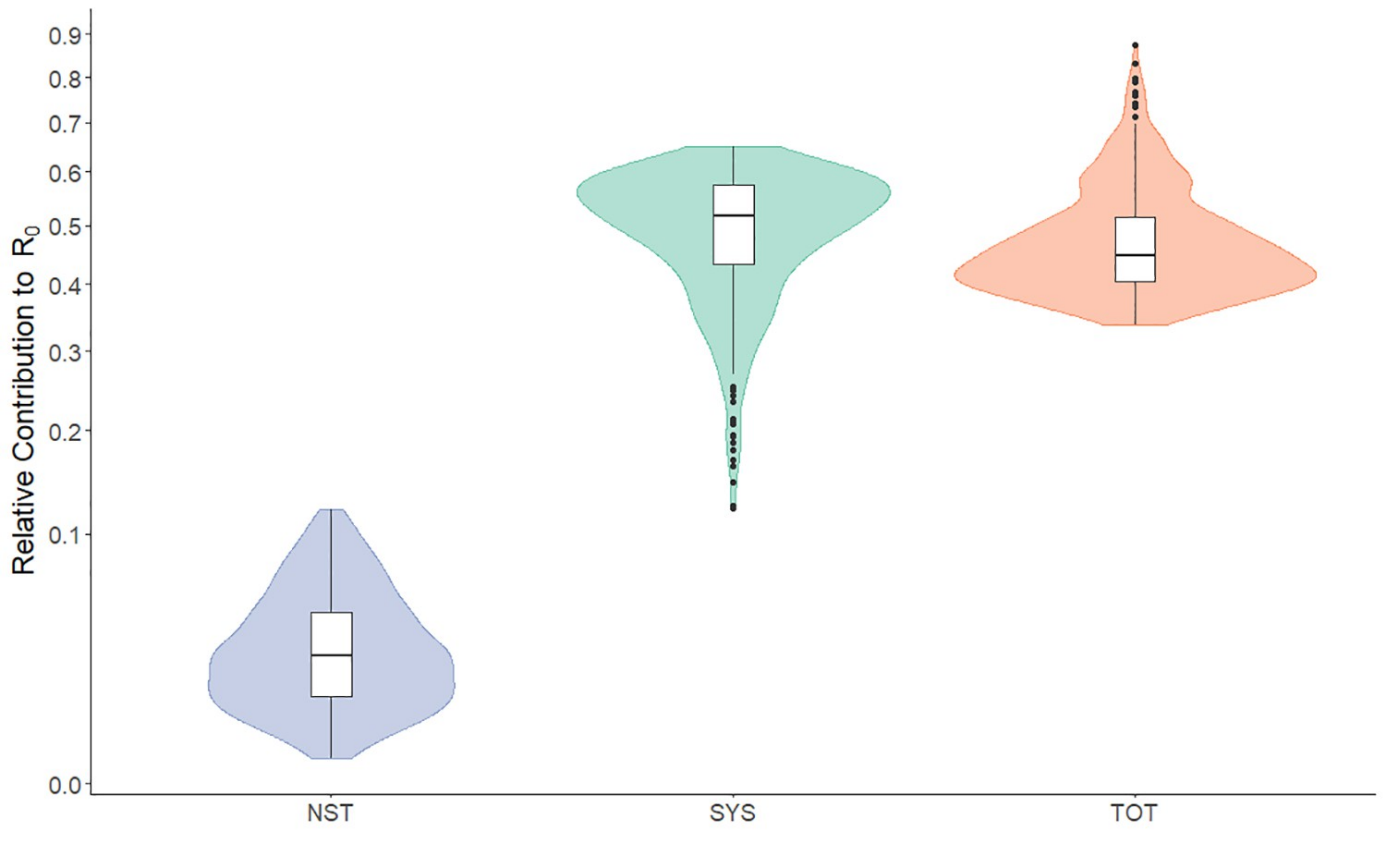
Distribution of values for relative contribution to R_0_ for each route of transmission across all scenarios and different scaling factors with transovarial transmission included in the model. (NST = Non-systemic transmission, SYS = Systemic transmission,TOT = transovarial). For results broken down by habitat type and scaling factor see Fig D in S2 Text. Coloured density kernels around boxplots show the distribution of R_0_ estimates from all model runs.

Across all models and scenarios, we found that the relative contributions to *R*_*0*_ for systemic transmission (Mean composite elasticity = 0.48, SD = 0.11) and transovarial transmission (0.46, SD = 0.08) were quite similar, and much higher than for non-systemic transmission (0.04, SD = 0.04) (Fig 3 and Fig D in S2 Text). In the model without transovarial transmission, almost all transmission was systemic (Mean composite elasticity = 0.965, SD = 0.04), with a very small contribution to *R*_*0*_ from non-systemic transmission (Mean composite elasticity = 0.03, SD = 0.04) (Fig F in S2 Text).

**Fig 4:**
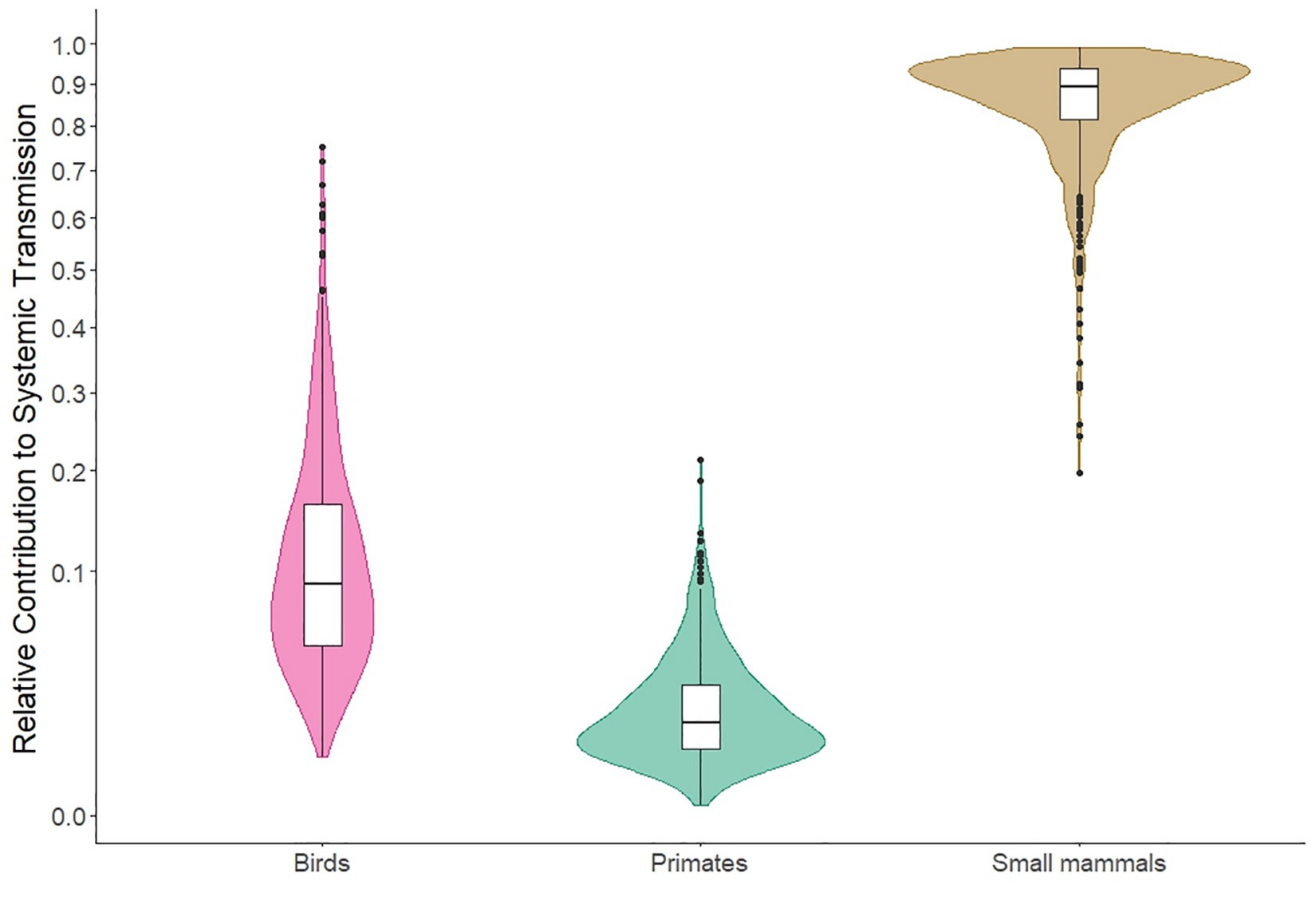
Distribution of values for relative contribution to systemic transmission for each host across all scenarios and different scaling factors with transovarial transmission included in the model. For results broken down by habitat type and scaling factor see Fig E in S2 Text. Coloured density kernels around boxplots show the distribution of R_0_ estimates from all model runs.

By further breaking down systemic transmission, it is clear that there is a marked difference between the relative contribution of small mammals, birds and primates to systemic transmission in both models (Fig 4 and Fig E in S2 Text). Small mammals had the greatest contribution to systemic transmission (Mean relative contribution to systemic transmission = 0.85, SD = 0.12) followed by birds (Mean relative contribution to systemic transmission = 0.12, SD = 0.11) and finally primates (Mean relative contribution to systemic transmission = 0.02, 0.02).

We assessed the relative contribution of each individual matrix element by examining the elasticity of individual matrix elements. This revealed that matrix elements that represent the number of eggs infected by a tick infected as a nymph and the number of eggs infected by an adult tick that was infected by as an egg (i.e. from their mother) were among the matrix elements that had the highest relative contribution to *R*_*0*_. The number of small mammals infected by ticks-infected-as-eggs and the number of nymphs infected by small mammals also had a high relative contribution to *R*_0_. (Table 2).

**Table 2:** Table shows the relative contribution to R_0_ from individual matrix elements (mean with 2.5% and 97.5% quantiles) and description of the contribution of matrix elements to number of infecteds for both the model using cattle density as a scaling factor and habitat type as a scaling factor. Matrix elements with a contribution of above 0.01 are included.

Matrix index	Description	Cattle Density	Habitat
k13	Number of eggs infected by tick-infected-as-nymph	0.23 (0.18–0.29)	0.22 (0.17–0.27)
k51	Number of small mammal infected by tick-infected-as-egg	0.2 (0.15–0.25)	0.21 (0.16–0.27)
k11	Number of eggs infected by tick-infected-as-egg	0.17 (0.08–0.26)	0.19 (0.09–0.27)
k35	Number of nymphs infected by an infected small mammal	0.17 (0.11–0.23)	0.18 (0.12–0.24)
k12	Number of eggs infected by tick-infected-as-larvae	0.05 (0.03–0.08)	0.05 (0.02–0.08)
k31	Number of nymphs infected by tick-infected- as-egg	0.05 (0.01–0.08)	0.03 (0.01–0.04)
k25	Number of larvae infected by an infected small mammal	0.03 (0.01–0.06)	0.03 (0.01–0.05)
k61	Number of birds infected by tick-infected-as-egg	0.03 (0.01–0.05)	0.03 (0.01–0.04)
k26	Number of larvae infected by an infected by bird	0.02 (0–0.03)	0.01 (0–0.02)
k14	Number of eggs infected by a tick-infected-as-adult	0.01 (0–0.02)	-
k36	Number of nymphs infected by an infected bird	0.01 (0–0.02)	0.01 (0–0.02)

**Fig 5:**
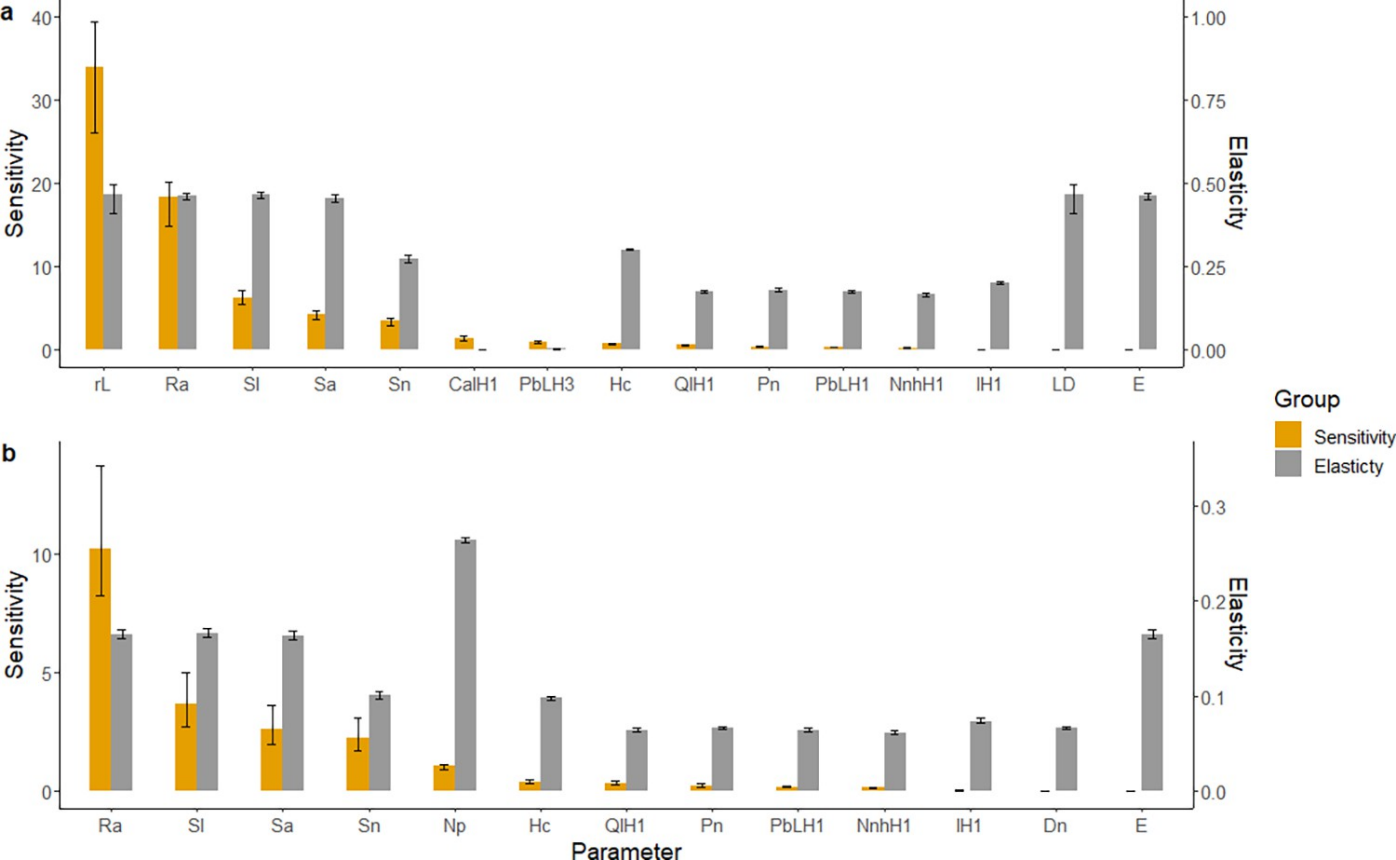
Mean sensitivity and elasticity values for individual parameters across all scenarios when transovarial transmission is included in the model. (a) Model with cattle density as scaling factor. (b) Model with habitat type as scaling factor. Error bars show 2.5% and 97.5% quantiles. Parameters were plotted if they had a sensitivity of greater that 1 or an elasticity of greater than 0.1.

The sensitivity of *R*_0_ to individual parameters was assessed for all models including transovarial transmission. Our analysis demonstrated high sensitivity and elasticity values for parameters within the models indicating that changes in some parameters can result in large changes to *R*_0_ (Fig 5). The rate of transovarial transmission (*R*_*a*_) had both high sensitivity and elasticity values. For the model with cattle density as a scaling factor, this analysis revealed relatively high sensitivity and elasticity values for *r*_*L*_ (the rate of increase in tick abundance and burden with cattle density), *S*_*l*_ (survival probability from egg to feeding larva), *S*_*n*_ (survival probability from feeding larva to feeding nymph) and *S*_*a*_ (survival probability from feeding nymph to feeding adult). These parameters also had relatively higher sensitivity values in the model using habitat type as a scaling factor with *S*_*l*_, *S*_*n*_ and *S*_*a*_ having relatively high values for sensitivity and elasticity values followed by *N*_*p*_ (1 –the proportion decrease in tick abundance).

## Discussion

This study developed a novel next generation matrix-modelling framework with wide applicability for improving understanding of the relative role of different hosts and transmission processes in the establishment of multi-host tick-borne pathogens in diverse and changing landscapes. When applied to Kyasanur Forest Disease in south India, despite relatively sparse empirical data, this approach highlights that key transmission processes (transovarial transmission) and reservoir hosts (birds and small mammals) likely play the most important role in maintaining transmission across all habitat types regardless of the scaling factor used. We thus recommend that the focus of research and management is extended beyond primates to identify other competent hosts that may play a more important role in maintaining KFD and influencing the distribution and density of infected ticks. This study also suggests potential variation in disease transmission risk between habitat types in the mosaic when habitat variation in tick abundance, tick burden and host density are accounted for. However, currently there is no available data outlining how tick abundance and burden on hosts varies for KFD-affected areas. Future refinement of models for this system will require more research on how tick abundance and burden on hosts varies in different habitat types in agroforest mosaics as well as investigation of the role of cattle in influencing tick abundance and distribution. Here we set our findings in the context of prior empirical and modelling work for this and other tick-borne disease systems and identify key research priorities to inform interventions to reduce the burden of KFD in India.

Based on our findings and those of Burthe et al. [7] it is becoming increasingly clear that there is a need to better assess the host preferences of *Haemaphysalis* ticks, host competence and susceptibility and subsequently the role of different hosts in influencing the maintenance of KFD and distribution of ticks. Understanding this at the local scale in areas prone to KFD outbreaks is also important as host range reported over larger regions may not necessarily translate to how ticks utilise hosts in a local context [60]. Much of the research on KFD and subsequent management recommendations have focused on the role of primates in creating hotspots for the transmission of KFD, with a focus on burning monkey carcasses and dusting insecticides within 50 feet of reported monkey deaths [7]. However, when accounting for both host density and tick burden on different hosts, our results suggest that small mammals and birds are likely to have the largest contribution to systemic transmission of KFD. Small mammals are an ideal reservoir for tick borne pathogens as an easily infected abundant host, which may not suffer from severe symptoms and regularly produces naive individuals [61]. Indeed, small mammals have already been highlighted as important reservoirs for several other tick borne pathogens with complex, multi-host transmission cycles including Lyme disease [62], Rickettsial pathogens [63] and other tick-borne flaviviruses such as tick-borne encephalitis [64]. Ground frequenting birds may also play an important role as an additional abundant and highly mobile host that could also introduce infected ticks to new areas [65–68] and a phylogeographic analysis has already indicated the possible role of migratory birds in transporting KFD infected ticks between Saudi Arabia and India [32,65,66]. Currently there is a lack of more recent data on the tick infestation of hosts and much of the available data on tick infestation of small mammals, birds and primates is from research conducted before 1972, after which further human modification of landscapes and habitats has taken place. Therefore, we need more up to date data on the distribution of potential hosts and on the tick burdens and the prevalence of KFD in small mammals, birds and primates to understand the contribution of these different hosts to maintenance of KFD and the distribution of ticks.

Additionally, we need a better understanding of the evolutionary relationships between KFD strains infecting these hosts versus those infecting humans in an effort to narrow down links between KFD strains infecting humans and strains circulating in wildlife reservoirs. Phylogenetic studies have regularly shown that pathogen species once considered to be generalists can exhibit cryptic host specificity, with individual strains exhibiting strong host associations, either as a result of compatibility barriers or barriers to encountering hosts [69]. For example, there is evidence of limited cross-species transmission of rabies lineages in bats, and strong host associations of *Borrelia burgdorferi* strains circulating in bank voles and chipmunks in France and for four *Anaplasma phagocytophilum* ecotypes circulating in vertebrate hosts in The Netherlands and Belgium [2,70]. Evidence from a study investigating KFD strains infecting primates, humans, ticks and rodents from the genus *Rattus* has so far demonstrated little divergence between strains infecting these different hosts, indicating a lack of host associations, however, further studies would help to elucidate the links between strains circulating in other wildlife reservoirs and strains infecting humans. Another recent study has also provided insights into the evolutionary relationships among KFD strains found in ticks, primates, and humans, as well as the likely pattern of KFD spread in Karnataka since 1957 [71]. In this study, the spread of KFD in Karnataka is attributed to the movement of tick-infested primates moving through forests, however, as of yet there is no information on the evolutionary relationships between human and primate strains and strains infecting small mammals, birds and ticks on cattle. Once again, further investigation is needed to understand the spread of KFD in India since it is just as plausible that longer distance dispersal of ticks infected with KFD could occur through movements of birds or cattle [32,65,66]. Dispersal by cattle could feasibly be subject to interventions aimed at limiting spread to new areas (e.g. use of repellents and pre- and post-movement tick removal on cattle) [7]. Ultimately, more up to date ecological and molecular studies will help to narrow down the key small mammal, bird, and tick species involved in maintenance of KFD and will be critical in refining future models. This would allow the behaviour and distribution of different tick and host species and tick related parameters, such as host preferences of different tick species, to be incorporated into future models.

Consistent with previous work and unusually among well-studied tick-borne pathogens [44], our models suggest that transovarial transmission plays an important role in the maintenance of KFD with a marked decrease in *R*_*0*_ (below 1) when transovarial transmission is excluded. Despite being demonstrated in laboratory studies using *Ixodes petauristae* and *Haemaphysalis spinigera* [27,28] there is still a lack of evidence that transovarial transmission of KFD occurs in the wild in *Haemaphysalis* ticks. However, the models used in this study suggest that ticks can act as a reservoir for KFD and highlight the importance of small mammals being infected by larvae infected via transovarial transmission, when transovarial transmission occurs. Whether or not ticks do act as a reservoir is an important question for understanding the maintenance of KFD as our sensitivity analysis highlights that *R*_*0*_ has a high sensitivity to the rate of transovarial transmission and also survival of different tick life stages. If ticks do play a significant role in maintaining KFD, cattle may play an important role by acting as widely available reproductive hosts for female adult ticks and influencing abundance and distribution of ticks. A number of other studies have demonstrated a role of larger mammals in tick-borne disease systems in Europe, showing the important role of deer in influencing abundance and distribution of *I*. *ricinus* in Europe [50,72,73] and the potential for non-systemic transmission through co-feeding on sheep (a non-competent host) in maintaining *B. burgdorferi [74]*. Currently, there is very little empirical research on how cattle density and habitat use might influence tick abundance and distribution. The limited empirical data on the number and life stage of ticks parasitising cattle in KFD-affected regions has also been largely collected in agricultural settings [31] rather than in the specific agro-forest mosaics in which spillover is occurring and where cattle are grazed across multiple habitat types. Due to this lack of data, we have assumed that an increase in tick abundance as cattle density increases would be similar to the rate of increase identified by existing research on deer densities and tick abundance in Europe. When looking at the sensitivity of *R*_*0*_ to the rate of increase in tick abundance due to cattle density it is clear that the model including cattle density as a scaling factor had high sensitivity to this parameter. This highlights the need to determine how cattle density and movement influences abundance and distribution of ticks to which humans are exposed to inform future models. This would be facilitated by sampling of ticks across different habitat types at the human-animal-forest interface and linking this to frequency of use of these habitat types by cattle and humans.

Our model had relatively high sensitivity to the habitat scaling factor compared to other parameters included in the model. It is well established that the survival, abundance and distribution of ticks is dependent on the overlap of suitable off-host environmental conditions, such as temperature and humidity, and the distributions and movements of hosts [75–77]. Numerous studies in Europe have found that habitat type or composition can influence the abundance of different tick species and the prevalence of associated pathogens such as *B*. *burgdorferi* and *Anaplasma phagocytophilum* [49,78–80]. Our models did show some variation in *R*_*0*_ across different land use classes when we assume that tick abundance, burden on hosts and host density varies with habitat type. These findings suggest that forested areas are more likely to have higher numbers of infected ticks and vertebrate hosts and subsequently may be areas of higher risk for people than areas of agriculture and plantations, paddy and human habitation. This is consistent with previous findings that people who regularly visit forests, graze their cattle in forests and handle dry leaves are at higher risk of contracting KFD in India [81]. However, it should be noted that our model is currently informed by the relative abundance of *Ixodes ricinus* ticks in different land use types in Europe [49] because we still have a limited understanding of how the abundance of *Haemaphysalis* ticks in KFD-affected areas varies across habitat types. It is understood that this genus of ticks generally prefers forested habitats [82] but our understanding of how human modifications to landscapes have altered suitability for ticks across landscapes is still limited and tick bite exposure may not necessarily be limited to the primary habitats of ticks [83]. A better understanding of risk in different habitat types can be gained by gathering empirical data on the abundance of questing ticks in these habitats and the prevalence of KFD in ticks. In particular, previous studies have highlighted evidence that KFD may be an ecotonal disease arising as a result of human encroachment into forested areas [33]. Modelling of historical outbreak patterns (2014–2020) indicates that spillover events to humans were more likely in diverse forest mosaics with high proportions of moist evergreen and plantation habitats [33]. Therefore, sampling tick populations in different habitats would ideally be accompanied by sampling across ecotones, for example from forests across ecotones and into matrix habitat [16,76], to improve our understanding of how the abundance and distribution of ticks varies across agro-forest mosaics. Ideally, this data should also be combined with data on human use of landscapes and coping capacity to shed light on how tick bite risk varies across focal areas [83].

Non-systemic transmission through co-feeding has been highlighted as playing an important role in the maintenance of flaviviruses such as tick-borne encephalitis virus in Europe [44,84]. In contrast, our model currently suggests that this form of transmission has a relatively low contribution to *R*_*0*_ for KFD, consistent with previous modelling of this system by Matser et al. [44]. The efficiency of non-systemic transmission can be dependent on the distance between feeding ticks, with efficiency shown to drop at distances of greater than 1cm (between co-feeding individuals) for *B. burgdorferi [85]* and the common aggregation of ticks within distances of around 1cm being thought to be important for transmission of tick borne-encephalitis [86]. Seasonality of activity of immature tick life stages can also influence non-systemic transmission between life stages, with seasonal synchrony of the activity of larvae and nymphs thought to be important in the transmission of TBEV in Europe [87]. In Karnataka, previous data from blanket drags and the prevalence of ticks on small mammals suggests that larval ticks are most abundant in November and December whilst nymphs are most abundant in January and February [46], highlighting potential for lack of synchrony for immature life stages and estimates for aggregation of ticks on hosts are not available for this system at this time. However, collection of up to date data on seasonality of different tick species, and their frequency and aggregation on hosts is ongoing, which will allow a better understanding of the potential contribution of non-systemic transmission to the maintenance of KFD.

The framework presented in this study can be applied to other tick-borne disease systems to examine the relative role of different transmission routes and hosts. It can also aid in prioritising future research to reduce uncertainty about the processes and mechanisms involved in the maintenance of tick-borne pathogens. Using this novel next generation modelling approach, we have highlighted a number of critical knowledge gaps that, if filled, would greatly improve our understanding of the ecology and maintenance of KFD in the context of agro-forest mosaics. There is a great need to improve our knowledge of the spatio-temporal distribution of ticks and hosts within these modified landscapes and to better understand how ticks are likely to utilise these hosts. Coupling this with information on prevalence of KFD and understanding the evolutionary relationships between any KFD variants will be crucial to developing improved mechanistic modelling approaches that can be used to link human use of landscapes with natural foci of this pathogen for informing effective intervention strategies.

## Supporting information

S1 Text Parameters, equations and data used in NGM models. (DOCX)

S2 Text Additional results from NGM models. (DOCX)
